# Editorial: Women in molecular innate immunity

**DOI:** 10.3389/fimmu.2023.1234710

**Published:** 2023-07-11

**Authors:** Francesca Granucci, Laura Marongiu, Birgit Strobl

**Affiliations:** ^1^ Department of Biotechnology and Biosciences, University of Milano-Bicocca, Milan, Italy; ^2^ Department of Biomedical Sciences, University of Veterinary Medicine Vienna, Vienna, Austria

**Keywords:** woman in science, young scientists, innate immunity, infections, inflammation

Despite facing various challenges and barriers, women in science have made significant contributions throughout history. The achievements of important personalities, like Marie Curie or Rosalind Franklin, have paved the way for future generations of women in scientific fields. Their example continues to inspire women in scientific disciplines around the world. Several factors contribute to the underrepresentation of women in different scientific fields. These factors include, for instance, societal stereotypes that often associate science with male characteristics, creating a perception that men are more naturally inclined or suited for science; the scarcity of women models; the lack of supportive policies like inadequate family leave policies, limited access to affordable childcare, and inflexible work environments, making it challenging for women scientists to manage both their personal and professional responsibilities; the demanding nature of scientific careers that can create challenges for women who also want to balance family responsibilities; the presence of unconscious bias in education that favor gender disparities, especially in science and affect encouragement and support given to girls in pursuing scientific subjects.

Progress has been made over the years in addressing these obstacles, but there is still work to be done to achieve gender equality in science.

With this special Research Topic, the section of Molecular Innate Immunity of Frontiers in Immunology offered to young women scientists the opportunity to promote their work by publishing original works or reviews addressing compelling problems in Molecular Innate Immunity.

The present Research Topic covers six publications that address different aspects of innate immunity.

The work presented by Agostinis et al. addresses the question of how SARS-CoV-2 infection impacts pregnancy, given the vertical transmission of the SARS-CoV-2 and the observed higher incidence of preeclampsia, preterm birth, caesarian section, and fetal mortality in infected mothers. The authors tested the expression of the three major receptors of SARS-CoV-2 by placental cells from infected and non-infected mothers and found that negative placentae were non-permissive to infection but the presence of SARS-CoV-2 altered the expression of receptors facilitating infections. Moreover, the Spike protein was capable of inducing the release of proinflammatory cytokine, trophoblast apoptosis and increased vascular permeability, all events compatible with pre-eclampsia-like syndrome.

Keeping on elucidating the molecular events that occur during infections, Tapia et al. investigated whether and how the human collectin SP-A regulated the activity of the cathelicidin peptide LL-37. The authors show that SP-A and a trimeric recombinant fragment thereof (rfhSP-A) interact with LL-37 and strongly reduce its tissue pathologic and inflammatory activity without reducing its anti-microbial function. Through this mechanism SP-A contributes to the maintenance of tissue integrity during lung infection.

Anti-inflammatory properties of complement factors, like C1s, have been described by (Lorvellec et al.). They showed that, under particular circumstances, the alarmin HMGB1 acquires specific tridimensional structures that favor the exposure of C1 cleavage sites. An N-terminal fragment obtained by C1s-mediated cleavage inhibits the activation of macrophages induced by the lipopolysaccharide (LPS). This work highlights an important and overlooked anti-inflammatory feedback loop.

The review presented by Duchesne et al. addresses the problem of asthma as an inflammatory response induced by alarmin cytokines, like interleukin (IL)-33, IL-25 and thymic stromal lymphopoietin (TSLP). The authors discuss the mechanisms that lead to the production of these alarmins from the airway epithelium and how the release of these factors and the interaction with their receptors can, in different circumstances, lead to the development of type 2 immunity, inflammation and allergic diseases.

Vuillier et al. elucidated, with their original work, that miR-3614-5p, a product of the TRIM25 gene, is induced by type I interferons (IFNs-I) in human immune and non-immune cells, represses the expression of the RNA-editing enzyme adenosine deaminase acting on RNA 1 (ADAR1) and lowers A-to-I editing of endogenous dsRNA. Mechanistically, authors show that miR-3614-5p directly targets the 3’ UTR of ADAR1 and that both miR- 3614-5p and ADAR1 transcripts are recruited to the RNA silencing complex (RISC) upon IFN-I stimulation. The study provides important new insights into the complex mechanisms that control ADAR1 activity and places miR-3614-5p into the molecular network that regulates innate immune sensing of dsRNA.

Finally, Veronique Collin-Faure et al. show the important toxic effect generated by nanoplastics. Using polystyrene nanoplatistics in the range of 100 nm to 6 microns the authors were able to demonstrate that polystyrene nano and microbeads alter the typical activities of macrophages like oxidative stress, expression of different surface molecules known to regulate immune responses, and functions of lysosomes and mitochondria. Supra-micron plastic particles were more dangerous than sub-micron particles. Therefore, the internalization of polystyrene alters the phenotype of macrophages and possibly innate immune responses.

This Frontiers in Immunology’ platform highlights the achievements and contributions of women in biology, thus helping to promote gender equity and challenging gender stereotypes and biases.

## Author contributions

All authors listed have made a substantial, direct, and intellectual contribution to the work and approved it for publication.

